# Supplementary material for: Occupational Radiation Exposure and Validity of National Dosimetry Registry among Korean Interventional Radiologists
Source: Int J Environ Res Public Health. 2021 Apr 15;18(8):4195. doi: 10.3390/ijerph18084195 (PMC8071388; doi:10.3390/ijerph18084195)
Supplement: Supplementary file 1 [file ijerph-18-04195-s001.zip › ijerph-1134272-supplementary.pdf]

Table S1. Validity analyses between the actively monitored doses and the NDR (national dose registry) doses of interventional radiologists during a quarter by amount of the time the badges were worn

Badge-wearing	N	Spearman's correlation coefficient (*p* value)		Dose difference	Paired t test p value
100% of time wearing a badge	26	0.39	(0.048)	0.43 ± 1.61	0.187
75-99% of time wearing a badge	11	0.50	(0.117)	-0.13 ± 0.75	0.567
25-74% of time wearing a badge	5	-0.40	(0.510)	0.16 ± 0.34	0.361
1-24% of time wearing a badge	4	-	-	7.47 ± 5.87	0.084
0% of time wearing a badge	10	-0.10	(0.793)	1.48 ± 1.39	0.008

Table S2. Validity analyses between the actively monitored doses and the NDR (national dose registry) doses among interventional radiologists who wore badges regularly, by job characteristics

Job characteristics	N[1]	Spearman's correlation coefficient (*p* value)		*p* value[2]
Calendar year began working as an interventional radiologist				0.925
<2007	18	0.36	(0.143)	
≥2007	19	0.39	(0.100)	
Years worked as an interventional radiologist				0.610
<10 years	17	0.48	(0.050)	
≥10 years	20	0.33	(0.156)	
Using ceiling-suspended shielding				0.359
≥75%	13	0.66	(0.014)	
<75%	23	0.41	(0.050)	
Using table-suspended shielding				0.638
≥75%	22	0.51	(0.016)	
<75%	14	0.36	(0.200)	

[1] Numbers may not add up to total due to missing information; [2] p value obtained from the test whether two correlation coefficients were equal to each other.
